# Insight into the roles of selection in speciation from genomic patterns of divergence and introgression in secondary contact in venomous rattlesnakes

**DOI:** 10.1002/ece3.2996

**Published:** 2017-04-23

**Authors:** Drew R. Schield, Richard H. Adams, Daren C. Card, Blair W. Perry, Giulia M. Pasquesi, Tereza Jezkova, Daniel M. Portik, Audra L. Andrew, Carol L. Spencer, Elda E. Sanchez, Matthew K. Fujita, Stephen P. Mackessy, Todd A. Castoe

**Affiliations:** ^1^ Department of BiologyThe University of Texas at ArlingtonArlingtonTXUSA; ^2^ Department of Ecology and Evolutionary BiologyUniversity of ArizonaTucsonAZUSA; ^3^ Museum of Vertebrate ZoologyUniversity of CaliforniaBerkeleyCAUSA; ^4^ National Natural Toxins Research Center and Department of ChemistryTexas A&M University KingsvilleKingsvilleTXUSA; ^5^ School of Biological SciencesUniversity of Northern ColoradoGreeleyCOUSA

**Keywords:** adaptation, divergence, gene flow, genomic clines, hybridization, RADseq

## Abstract

Investigating secondary contact of historically isolated lineages can provide insight into how selection and drift influence genomic divergence and admixture. Here, we studied the genomic landscape of divergence and introgression following secondary contact between lineages of the Western Diamondback Rattlesnake (*Crotalus atrox*) to determine whether genomic regions under selection in allopatry also contribute to reproductive isolation during introgression. We used thousands of nuclear loci to study genomic differentiation between two lineages that have experienced recent secondary contact following isolation, and incorporated sampling from a zone of secondary contact to identify loci that are resistant to gene flow in hybrids. Comparisons of patterns of divergence and introgression revealed a positive relationship between allelic differentiation and resistance to introgression across the genome, and greater‐than‐expected overlap between genes linked to lineage‐specific divergence and loci that resist introgression. Genes linked to putatively selected markers were related to prominent aspects of rattlesnake biology that differ between populations of Western Diamondback rattlesnakes (i.e., venom and reproductive phenotypes). We also found evidence for selection against introgression of genes that may contribute to cytonuclear incompatibility, consistent with previously observed biased patterns of nuclear and mitochondrial alleles suggestive of partial reproductive isolation due to cytonuclear incompatibilities. Our results provide a genome‐scale perspective on the relationships between divergence and introgression in secondary contact that is relevant for understanding the roles of selection in maintaining partial isolation of lineages, causing admixing lineages to not completely homogenize.

## 1 Introduction

Understanding the process of speciation requires insight into both the processes that underlie lineage divergence in isolation and the processes that maintain lineage integrity (i.e., limit gene flow) in the face of gene flow during secondary contact. Evolutionary divergence involves both selection‐driven and neutral changes as lineages evolve (Coyne & Orr, 2004; Kuehne, Murphy, Francis, & Sniegowski, 2007; Nosil, Funk, & Ortiz‐Barrientos, 2009). Loci that have accumulated divergence neutrally in allopatry may eventually act to limit gene flow between sister lineages due to a reduction in hybrid fitness when introgression occurs (e.g., cytonuclear incompatibilities; Orr, 1995; Ulloa, Corgan, & Dunford, 1995; Fishman & Willis, 2006; Sambatti, Ortiz‐Barrientos, Baack, & Rieseberg, 2008; Gagnaire, Normandeau, & Bernatchez, 2012). Conversely, locally adapted genes subject to geographically variable selection may also contribute to reduced gene flow and genetic isolation in structured populations that come into secondary contact (Boughman, 2001; Nosil, Egan, & Funk, 2008; Orr & Smith, 1998; Rosenblum, 2006), as alleles from one parental population may offer a greater fitness advantage to hybrids. Accordingly, insight into the roles of selective and neutral evolutionary processes in driving speciation can be gained from the relationships (or lack thereof) between genes important in divergence and introgression in natural systems.

While links between adaptive evolution and speciation have been established (Faria et al., 2014; Schluter & Conte, 2009), a largely unanswered question is whether the same genomic regions are important in both the process of divergence in isolation (i.e., loci under divergent selection and adaptation) and in preventing gene flow in secondary contact (i.e., loci underlying partial or complete reproductive isolation). This is an important question central to our understanding of how speciation proceeds (or is reversed), as there is a wealth of examples of secondary contact and hybridization in diverse taxa (Payseur & Rieseberg, 2016). Despite this, there are few studies that have specifically examined adaptive evolution in both divergence and in secondary contact (e.g., Gompert et al., 2012a; Nosil, Parchman, Feder, & Gompert, 2012; Parchman et al., 2013). These studies have explored connections between divergent selection during isolation and selection against introgression upon secondary contact, and provide evidence that loci evolved under divergent selection also contribute to partial or complete reproductive isolation during hybridization due to deleterious fitness effects on hybrids. However, these studies are limited to a small representation of taxa (two insect systems and one bird system), and it is unknown how broadly the genomic relationships between divergence and admixture processes are found in nature. There is thus a need to test the consistency of these relationships broadly across taxa to appreciate their evolutionary significance, and how such relationships may be dependent on particular taxa or on the degree of differentiation between diverged lineages.

**Figure 1:**
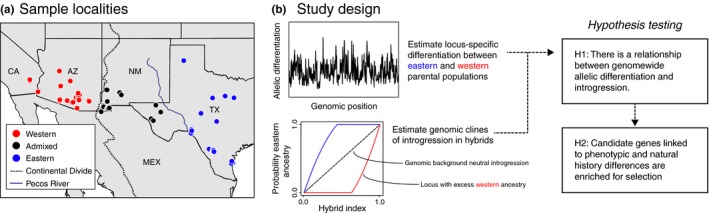
Sampling and study design. (a) Map of sampled localities, where red, blue, and black circles represent western, eastern, and admixed populations, respectively. The Continental Divide is depicted as a dashed line and the blue line represents the Pecos River. (b) Schematic view of the study design in this paper. The first steps were to estimate allelic differentiation between parental populations and genomic introgression within hybrids to identify outlier selected loci. In the lower panel, the probability of parental ancestry is compared to the hybrid index of the admixed population. Here, the dashed line represents the expectation of neutral introgression. The blue and red lines represent loci with evidence of excess eastern and western ancestry, respectively, due to the action of selection. Allelic differentiation and introgression estimates were then compared to determine whether a genomewide relationship between divergence in allopatry and admixture upon secondary contact exists, and we tested whether genes linked to outlier loci were enriched for candidate gene sets of interest

In this study, we address this need by examining a previously described a hybrid zone between two divergent and historically isolated lineages of the Western Diamondback Rattlesnake (*Crotalus atrox*) (Castoe, Spencer, & Parkinson, 2007; Schield et al., 2015). *C. atrox* inhabits a broad distribution including regions of the Chihuahuan and Sonoran deserts, as well as the adjacent temperate grasslands in the southwestern United States and northern Mexico (Campbell & Lamar, 2004). The range of *C. atrox* is bisected by the Continental Divide (Figure 1a), which represents a major constriction of two large tracts of occupied habitat to the east and west. The two distinct lineages (i.e., parental populations) of *C. atrox* generally coincide with populations east and west of the Continental Divide, and the admixture region between these lineages is bounded by the Continental Divide to the west and the Pecos River to the east; this admixture region is generally dominated by Chihuahuan Desert habitat (Castoe et al., 2007; Schield et al., 2015), and we here refer to this area as the Chihuahuan region. Additionally, ecological niche models projected onto the climatic conditions of the last glacial maximum suggest that ancestral *C. atrox* populations diverged in prolonged isolation and have since converged within the admixture region that exists geographically between their two ancestral ranges (Schield et al., 2015). Using a comparison of mtDNA and nuclear SNPs, we identified an asymmetry in the proportions of nuclear alleles paired with mitochondrial haplotypes in the Chihuahuan region. Specifically, we did not observe individuals with largely “western” nuclear genomes with “eastern” mitochondrial haplotypes (Schield et al., 2015). We proposed that this asymmetry could have arisen from cytonuclear incompatibilities that evolved during divergence in isolation and that reduce the fitness of hybrids in secondary contact, which we explore further in this paper with expanded data and analyses.

In addition to studying the possibility of cytonuclear incompatibility between historically isolated lineages of *C. atrox,* in this paper we consider aspects of their biology that may have been driven by adaptive evolution and lineage‐specific patterns of selection. Previous studies have explored phenotypic and natural history diversity across the range of *C. atrox* and, in particular, differences between eastern and western populations. For example, there are differences in body coloration and patterning between populations (Klauber, 1930; Spencer, 2003, 2008), with darker overall coloration and fewer body blotches found in the eastern population. Spencer (2003) also discovered differences in reproduction between eastern and western females; reproductive differences included a significant correlation between longitude and the number of follicles per female, with larger and more numerous follicles in western females, along with differences in offspring size. Finally, venom toxicity and composition are known to vary between eastern and western populations, including increased protease activity in the eastern population (Minton & Weinstein, 1986).

Our broad aim in this study was to identify genomewide patterns of selection in divergent lineages of *C. atrox*, and test whether these or different genomic loci were also under selection in zones of admixture where these lineages meet in secondary contact. To accomplish this, we first use our large nuclear locus dataset to test competing models of speciation in Western Diamondbacks to test the hypothesis of population divergence followed by secondary contact, which is strongly supported by previous studies (Castoe et al., 2007; Schield et al., 2015). We then leverage this system to address the following questions central to the role of adaptive evolution in lineage divergence and introgression in the process of secondary contact and hybridization: (1) Is there evidence for loci linked to genomic regions under divergent selection for local adaptation within parental lineages/populations? And (2) Is there evidence for loci under selection to resist introgression in the hybrid population? We then test the hypothesis that there is a genomic relationship (i.e., a positive correlation) between genetic differentiation in parental populations and introgression in secondary contact (Figure 1b). To address these questions, we analyzed patterns of variation in our genomewide sampling of nuclear SNPs from the hybrid and parental populations, and interpreted these using existing snake genomic resources. To explicitly test a null model of neutral drift across loci contributing to divergence between parental populations, we implement a newly developed method, *GppFst* (Adams, Schield, Card, Blackmon, & Castoe, 2016), to identify patterns of allelic differentiation that are poorly explained by neutral processes and more likely driven by selection. We used analyses of genomic clines implemented in *bgc* (Gompert & Buerkle, 2012) to study patterns of introgression in hybrids and identify genomic regions that resist introgression due to selection. This genomic cline approach has been used in several recent studies to explore patterns of introgression in hybrids (e.g., Caseys, Stölting, Barbará, González‐Martínez, & Lexer, 2015; Gompert et al., 2012a; Janousek et al., 2012; Nosil et al., 2012; Parchman et al., 2013; Trier, Hermansen, Sætre, & Bailey, 2014), and simulations have demonstrated that it is robust to the detection of loci with clines that deviate from the genomewide expectation (Gompert & Buerkle, 2011). Finally, to explore potential selective pressures driving patterns of divergence and introgression, we test the hypothesis that a priori sets of candidate genes are enriched for outlier loci (Figure 1b). A priori candidate gene sets were curated specifically to address two chief interests: (1) the observed asymmetry in complements of nuclear and mitochondrial alleles in admixed individuals that may be due to incompatibility between mitochondrial haplotypes and nuclear genomic background, and (2) prominent phenotypic and natural history differences between populations of *C. atrox* (i.e., venom composition, coloration, and reproductive biology).

## 2 Materials and Methods

### 2.1 Sampling design, RADseq data generation, and computational analysis

In a previous study (Schield et al., 2015), we generated restriction site‐associated DNA sequencing (RADseq) data for 43 samples from throughout the range of *C. atrox*. To increase sampling, we generated new RADseq data from 32 additional individuals including: 15 samples west of the Continental Divide, seven samples east of the west Texas mountains, and 10 samples from the Chihuahuan region (Figure 1a; Table S1). Sampling of these populations enabled the comparative approach illustrated in Figure 1b. We also generated RADseq data for six individuals of the Mohave Rattlesnake (*C. scutulatus*; Table S1), a close relative of *C. atrox*. These were used as the outgroup comparison for lineage delimitation analyses. New data generation followed Schield et al. (2015), which used a slightly modified version of the double‐digest RADseq approach of Peterson, Weber, Kay, Fisher, and Hoekstra (2012), targeting ~20,000 genomic loci per individual (see Appendix S1 for detailed methods). We removed PCR clones from raw sequencing reads using the Stacks clone_filter module (Catchen, Hohenlohe, Bassham, Amores, & Cresko, 2013), and trimmed the adapter sequence and UMI bases from filtered reads (see Appendix S1). Reads were demulitplexed to individuals using the Stacks process_radtags module. Trimmed paired reads were assembled into a consensus “pseudo‐reference” genome using the de novo assembly option in dDocent (Puritz, Hollenbeck, & Gold, 2014) to form contigs, using program defaults. This yielded 24,115 de novo *C. atrox* loci with an average length of 192 bases (consistent with our paired‐read length). Individual read files were mapped to the pseudo‐reference using the BWA‐mem algorithm (Li & Durbin, 2009), specifying paired‐read inputs, an open gap penalty of 3, and a mismatch penalty of 2. SAMtools v1.3 and BCFtools v1.3 (Li et al., 2009) were used to process mapping files and to call single nucleotide polymorphisms (SNPs). Variants were filtered using VCFtools (Danecek et al., 2011) to retain SNPs with quality scores above 30; to reduce linkage among variant sites, we sampled one variant site from each locus. We also filtered to remove SNPs with minor allele frequencies below 0.05, as these lack useful ancestry information for analyses of differentiation and introgression (Gompert et al., 2012a). We performed analyses of genes in proximity (i.e., genetically linked) to SNPs by inferring putative orthology between our pseudo‐genome contigs and the King Cobra genome (Vonk et al., 2013) using a BLAST search (Altschul, Gish, Miller, Myers, & Lipman, 1990). BLAST results were filtered by e‐value, and a single alignment with the lowest e‐value per variable contig was retained. Here, we assumed that genetic variation consistent with natural selection observed at RADseq loci is due to physical linkage to genic targets of selection. We used the King Cobra genome as a reference because it is currently the closest relative to *C. atrox* with a fully annotated genome sequence.

### 2.2 Two‐dimensional allele frequency spectra and competing models of divergence

We tested competing population genetic models of divergence between parental populations using δaδi to analyze two‐dimensional allele frequency spectra (2D‐AFS; Gutenkunst, Hernandez, Williamson, & Bustamante, 2009), and further investigated evidence for the presence of two distinct ancestral lineages using Bayesian lineage delimitation implemented in BFD* (Leache, Fujita, Minin, & Bouckaert, 2014). Rather than the pipeline detailed above for downstream divergence and introgression analyses, here we used the specifications detailed in Schield et al. (2015) to generate input data because we leveraged existing workflow (Portik et al., In Review; https://github.com/dportik/dadi_pipeline) that uses Stacks (Catchen et al., 2013) output to format data for analyses in δaδi. In brief, loci were filtered in Stacks to retain loci only if they were present in both parental populations and represented by a minimum of 30% of individuals at a minimum coverage depth of 6×. This pipeline yielded 3,756 loci for analyses of demographic models and lineage delimitation.

Using δaδi, the folded 2D‐AFS was generated from the nuclear SNP data for a maximum of 23 individuals from the western population and 16 individuals from the eastern population. We excluded admixed individuals and individuals near the hybrid region to infer long‐term gene flow, rather than the effect of contemporary hybridization. To account for missing data, we projected down to smaller sample sizes (western: 24 alleles, eastern: 24 alleles), resulting in 2,646 segregating sites. Nine alternative demographic models were fit to the 2D‐AFS (Table 1; Fig. S1), including models with and without divergence. For each model, 20 sets of randomly perturbed parameters were optimized by the Nelder–Mead method for a maximum of 100 iterations. The 2D‐AFS was simulated from each parameter set, and extrapolation was performed with a grid size of [40,50,60]. Log‐likelihoods were estimated using the multinomial approach, and models were evaluated using the Akaike information criterion (AIC) based on the replicate with the highest log‐likelihood score.

**Table 1:** Results of population genetic model comparison using the two‐dimensional allele frequency spectrum (2D‐AFS) between western and eastern *C. atrox* populations

Model	AIC	∆AIC	RL	*w*_*i*_	log‐lik	params	theta	nu1	nu2	m12	m21	T1	T2
Divergence and isolation, secondary contact with symmetric migration	639.1	0.0	1	0.78	−314.6	5	93.8	1.01	3.54	0.189	m12	0.404	0.703
Divergence and isolation, secondary contact with asymmetric migration	642.4	3.3	0.192	0.15	−315.2	6	77.8	1.23	4.08	0.144	0.196	0.119	1.475
Divergence with symmetric migration	645.3	6.2	0.045	0.035	−318.6	4	122.4	0.74	2.77	0.184	m12	0.659	–
Divergence with ancient symmetrical migration, isolation	646.2	7.1	0.029	0.022	−318.1	5	77.6	1.17	4.15	0.310	m12	1.496	0.148
Divergence with ancient asymmetrical migration, isolation	647.6	8.5	0.014	0.011	−317.8	6	105.4	0.89	2.85	0.233	0.398	0.956	0.071
Divergence with asymmetric migration	662.6	23.5	0	0	−326.3	5	112.8	0.67	3.29	0.480	0.032	0.761	–
Divergence and isolation	683.5	44.4	0	0	−338.7	3	131.4	0.76	2.88	–	–	0.467	–
Growth model, no divergence	4498.4	3859.3	0	0	−2246.2	3	115.7	–	–	–	–	–	–
Neutral model, no divergence	4736.9	4097.8	0	0	−2368.4	0	232.5	–	–	–	–	–	–

**Figure 2:**
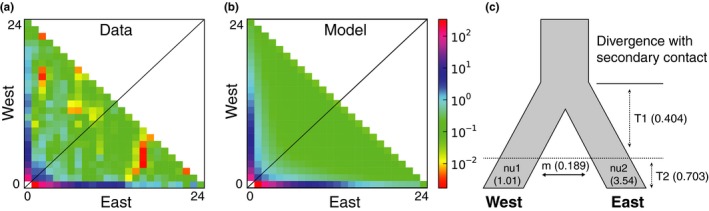
Summary of the two‐dimensional allele frequency spectrum (2D‐AFS) between parental populations and results of demographic model testing. (a, b) 2D‐AFS plots of empirical data and the inferred best‐fit model predictions, respectively. A legend of the spectrum of allele frequencies is shown to the right of b. (c) Inferred best‐fit model of population divergence in isolation followed by recent secondary contact, with demographic parameter estimates from δaδi. For details of parameter estimates and abbreviations, see Table 1

The best‐fit model and parameters are in bold. Visual comparison of the 2D‐AFS for the data and the best‐fit model is provided in Figure 2.

AIC, Akaike information criterion; RL, relative likelihood; *w*_*i*_, Akaike weight; params, number of parameters in model; theta, 4N_ref_μL; nu1, effective population size of western group; nu2, effective population size of eastern group; m12, migration rate from eastern population to western population; m21, migration rate from western population one to eastern population; T1, scaled time between population split and the present or T2, the scaled time of secondary contact or isolation interval.

We explicitly tested for the presence of two distinct *C. atrox* lineages using genomewide Bayes factor lineage delimitation implemented in BFD* (Leache et al., 2014). Similar to demographic model analyses, we tested two competing hypotheses of either two divergent parental lineages (east and west lineages, Model A) or a single combined *C. atrox* lineage (Model B), using *C. scutulatus* as an outgroup (see section 2.1). We generated a combined alignment of 8 *C. atrox* samples randomly sampled from both the eastern (*n* = 4) and western (*n* = 4) parental populations and an additional four outgroup samples (*C. scutulatus*) in Stacks, using the same specifications as above for δaδi input, which yielded a total alignment of 463 biallelic SNPs. We set a diffuse gamma prior (α = 1, β = 10) for the population parameters (θ) based on the average pairwise genetic distance (*average* π = 0.12) across all loci, and a diffuse gamma prior (α = 1, β = 23.8) on the speciation rate (λ) to reflect an expected genetic divergence between *C. atrox* and *C. scutulatus* inferred from previous studies (E[divergence] = 0.35 (Reyes‐Velasco, Meik, Smith, & Castoe, 2013)). Additionally, we fixed the backward and forward mutation rates equal to the observed frequencies within our dataset (μ = 2.85, υ = 0.61). We ran path sampling analyses for 48 steps, specifying α = .3, each for a total of 100,000 MCMC generations and set the burn‐in threshold to remove the first 10,000 generations.

We used STRUCTURE (Pritchard, Stephens, & Donnelly, 2000) to estimate admixture proportions across the SNP dataset. We ran STRUCTURE on nuclear SNPs under models of *K *=* *1–10 (three iterations each; 100,000 burn‐in generations, 900,000 sampled generations). We used the Δ*K* method (Evanno, Regnaut, & Goudet, 2005) implemented in StructureHarvester (Earl & Vonholdt, 2012) to determine the most likely number of *K* ancestral populations and visualized the Q‐matrix (posterior probabilities of ancestry proportions) spatially using the tessplot function (Jay et al., 2012) in R (R Development Core Team 2012).

### 2.3 Analysis of population genetic differentiation

We used three measures to summarize nucleotide diversity and to estimate genetic differentiation among loci between eastern and western parental populations. To understand the relative allelic diversity within each population, we first estimated Nei and Li's (1979) π across loci and then calculated the difference between π_eastern_ and π_western_ (referred to as Δπ_eastwest_ hereafter). Our expectation for Δπ_eastwest_ is that a locus with low diversity in both populations will yield a value near 0, while a locus with greater diversity in the eastern population than western will yield a positive value, and one with greater diversity in the western population than the eastern will return a negative value. We calculated relative population genetic differentiation between populations using Weir and Cockerham's *F*_ST_ (Weir & Cockerham, 1984). *F*_ST_ is a relative measure of divergence between populations, and may be influenced by differences in nucleotide diversity within populations (Cruickshank & Hahn, 2014); therefore, we also used an absolute measure of population differentiation, d_*xy*_, to characterize divergence between eastern and western populations, using the calculation described in Irwin, Alcaide, Delmore, Irwin, and Owens (2016). Weir and Cockerham's correction sometimes yields negative values of *F*_ST_, and we converted any negative estimates to zero (the lower bound indicative of panmixia at a given locus).

We designated outlier loci with greater‐than‐expected values of population differentiation using a multivariate approach, in the program MINOTAUR (Verity et al., 2016), using information from distributions of *F*_ST_, d_*xy*_, and Δπ_eastwest_ to identify loci that differ significantly from the genomic background. An advantage of this approach is that it makes no specific assumptions based on a single summary statistic or the demographic history of the populations, but rather uses the combined information provided by multiple statistics and is robust for detecting genomic outliers derived from a wide variety of evolutionary scenarios. We specifically used the Mahalanobis distance (MD, defined as the distance from the multivariate centroid; Mahalanobis, 1936) measure to delineate outlier loci where differentiation between parental populations has likely been driven by selection. Specifically, loci were considered statistical outliers if the locus‐specific MD exceeded the 95th quantile of the genomic distribution, and were considered strong outliers if they exceeded the 99th quantile.

We also wished to determine whether statistical outlier loci had estimates of allelic differentiation that would not be expected under a model of neutral evolution. To test this, we designed a software program, *GppFst* (Adams et al., 2016; https://github.com/radamsRHA/GppFst), which conducts posterior predictive simulations (PPS) of *F*_ST_ and d_*xy*_ under a strict model of evolution in the absence of selection. To conduct PPS analysis, we estimated divergence time and population parameters for the parental eastern and western populations using the 7,031 SNPs detailed above (τ_eastwest_, θ_west_, θ_east_, and θ_eastwest_) via Markov chain Monte Carlo (MCMC) sampling implemented in the program SNAPP (Bryant, Bouckaert, Felsenstein, Rosenberg, & RoyChoudhury, 2012). We stress that parameterization in SNAPP for *GppFst* was done using only structured parental populations and did not include admixed individuals. Additionally, we found very strong Bayes factor (BF) support for two distinct *C. atrox* lineages (BF = −336; see section 2.1); we would not expect strong support for this model in the BFD* framework if gene flow were pervasive between the eastern and western populations (Zhang, Zhang, Zhu, & Yang, 2011). The mean posterior estimates of population genetic parameters used for *GppFst* simulations from SNAPP were as follows: θ_east_ = 0.217, θ_west_ = 0.0592, θ_eastwest_ = 0.0615, and τ_eastwest_ = 0.00578. We ran MCMC for a total of 500,000 generations, sampling every 1,000 generations. We assessed posterior convergence and stationarity using Tracer (for all parameters ESS > 500; Drummond & Rambaut, 2007) and discarded the first 125,000 (25%) steps as burn‐in, leaving total of 375 MCMC samples used to generate a PPS *F*_ST_ distribution. For each MCMC step, we then simulated 100 independent loci with a length of 190 base pairs (equal to the average length of variant loci) under a JC69 model using the R package phybase (Liu & Yu, 2010) with random sampling of individuals from the empirical distributions of locus coverage for eastern and western samples, respectively.

We calculated *F*_ST_ and d_*xy*_ for a randomly sampled polymorphic site for each simulated locus to generate a theoretical distribution of values, which allowed us to compare empirical distributions of *F*_ST_ and d_*xy*_ to identify loci with levels of divergence that are poorly explained by the neutral model of divergence. Specifically, we calculated the probability that the proportion of empirical loci with a given value of allelic differentiation is observed in the posterior predicted distribution, and thus were able to reject a strict model of neutral evolution where this probability is very low. Additionally, the use of simulations allowed us to account for the potential influence of unevenness in sample sizes across loci and differences in effective population size between the two populations. We considered statistical outliers from multivariate outlier detection “positives” for selection if they were also poorly explained by a neutral model of evolution based on their comparison to simulated *F*_ST_ and d_*xy*_ distributions in *GppFst*.

### 2.4 Analysis of genomic introgression

We conducted Bayesian estimation of genomic clines using the program *bgc* (Gompert & Buerkle, 2011) to identify loci that defy neutral expectations of admixture compared to the genomic background. We segregated samples into western, eastern, and admixed populations (Figure 1), and calculated locus‐specific allele frequencies for each population. *bgc* estimates the hybrid index (*h*) for each individual in an admixed population, representing the proportion of an individual's genome inherited from one parental population. Hybrid index and two locus‐specific genomic cline parameters (α, the genomic cline center parameter; and β, the genomic cline rate parameter) are used to estimate the posterior probability of inheritance from one parental population (Φ) at a given locus within the admixed population. Under this model, if both α and β are equal to 0, *h* and Φ will be equivalent (Gompert & Buerkle, 2011), and will match the neutral genomic background expectation (dashed line in the genomic cline panel of the “study design” in Figure 1b). Loci enriched for selection can be identified based on each cline parameter and classified based on their locus‐specific cline parameters relative to a genomewide distribution (Gompert & Buerkle, 2011; Gompert et al., 2012a).

We used two methods to identify outlier loci that exhibit exceptional introgression compared to the neutral genomic background expectation. First, we considered loci outliers if they had evidence of excess ancestry from one parental population (i.e., the 95% confidence interval of the α parameter did not include 0; see Gompert & Buerkle, 2012) and also had a median α within the tails of the 95th quantile of the median α distribution. Second, “strong” statistical outliers were determined using the locus‐effect quantiles for α (γ‐quantile) relative to a genomewide distribution (Gompert & Buerkle, 2011); loci were considered strong outliers if their γ‐quantile fell outside of the interval *q*_*n*_, where $\frac{1-n}{2}<q_{n}<\frac{n}{2},$ where *n *=* *0.1. While both statistical outlier loci (from divergence analyses) and loci with evidence of excess ancestry are not definitively targets of selection, they do exhibit patterns that are poorly explained by neutral introgression and are often found in genomic regions impacted by strong selection (Gompert & Buerkle, 2012).

To compare empirical genomic clines to expectations of neutral introgression, we simulated a “null” admixed population by randomly sampling alleles observed in the empirical parental populations for each locus equal to the number of individuals in the empirical admixed population (*n *=* *21). Here allelic information per individual per locus was derived from two random draws from parental alleles (eastern or western) for that locus. To evaluate multiple random draws of parental alleles for each locus per individual, we repeated this process five times and ran parallel *bgc* analyses to establish a benchmark for expectations under neutral introgression using the specifications detailed in Appendix S1.

### 2.5 Comparative analyses of divergence and introgression

We tested the hypothesis that there is a genomewide relationship between allelic differentiation accumulated during divergence and patterns of introgression in secondary contact using linear correlation analyses. We compared locus‐specific univariate nucleotide diversity and allelic differentiation statistics (Δπ_eastwest_, *F*_ST_, and d_*xy*_) as well as the multivariate Mahalanobis distance from divergence analysis with the genomic cline center parameter, α. We examined these relationships using the absolute values of Δπ_eastwest_ and α.

We tested for significant overlap in genes linked to outliers from divergence and introgression analyses and for enrichment of candidate gene sets related to traits of interest by first determining the nearest up‐ and downstream annotated genes from each orthologous cobra genome region. We then built a distribution of quantities of overlapping genes based on random expectation by producing 1,000 randomly resampled datasets using the background list of all genes linked to sampled loci, and compared these to observed quantities of overlapping genes between outliers from divergence and introgression analyses.

We specified five a priori candidate gene sets of interest: snake venom genes, coloration genes, genes involved in reproductive output and timing, nuclear‐encoded mitochondrial proteins (nuc‐mt), and nuclear‐encoded subunits specifically involved in oxidative phosphorylation (nuc‐oxphos), and tested for enrichment of these candidate gene sets within the sets of genes we identified as being linked to outlier loci from divergence or introgression analyses using Fisher's exact tests. Venom, reproduction, and coloration candidate gene sets were chosen to enable us to explicitly test whether genes related to known phenotypic differences between *C. atrox* populations are highly differentiated and also contribute to reproductive isolation in hybrids. Nuc‐mt and nuc‐oxphos gene sets were curated to test for patterns of enrichment in divergence and introgression outliers because of their interactions and coevolution with the mitochondrial genome. Here, we were specifically interested if a decoupling of this coevolution due to introgression and incompatibility between nuclear and mitochondrial genomes could explain the previously observed asymmetry in complements of nuclear alleles with western and eastern mitochondria (Schield et al., 2015). For additional rationale behind these specific candidate gene sets, see the section 1, and see Appendix S1 for details of candidate gene BLAST set construction and analysis.

## 3 Results

### 3.1 Results of variant calling pipeline for divergence and introgression analyses

The assembled *C. atrox* de novo “pseudo‐reference genome” included 24,115 loci constructed from sequenced RADseq reads. Following mapping and filtering to remove indels, spurious base calls introduced by sequencing error, non‐biallelic sites, and multiple variants per locus, we retained 7,031 SNPs for divergence and introgression analyses. Orthologous regions from BLAST matching of *C. atrox* contigs to the cobra genome were fairly evenly spread across 3,832 cobra scaffolds, with a range of 1–20 (mean of 1.87) de novo contigs per cobra scaffold (cobra scaffold *N*_50_ = 226 Kb). While the proportion of missing data was variable across loci (0%–96%), we did not observe significant correlations between missing data and allelic differentiation statistics (*p *=* *.538) or genomic cline parameter estimates (*p *=* *.917).

### 3.2 Evidence for divergence in isolation and secondary contact of lineages

Analysis of the 2D‐AFS and competing population genetic models in δaδi indicated that models that did not include population splitting fit poorly to our data when compared to models that included lineage divergence. Untransformed parameters are provided in Table 1, along with an estimate of θ (θ = 4NrefμL, where L is the total length of sequenced region SNPs were ascertained from). A model of population divergence in isolation followed by recent gene flow in secondary contact provided the best fit our data (Figure 2a–c, Table 1), with minor residuals. This model was very strongly supported with an Akaike weight = 0.78, which fit the data substantially better than even the second best supported model of divergence with secondary contact and asymmetric gene flow (Akaike weight = 0.15). Akaike weights were negligible for all other models indicating essentially no support for any of these alternative scenarios (see Burnham & Anderson, 2003). Under the model of divergence with secondary contact, δaδi estimated greater effective population size and genetic diversity in the eastern population than in the western, consistent with previous studies comparing demographic parameters (Castoe et al., 2007; Schield et al., 2015). The best‐fit model of divergence with secondary contact also suggests equivalent migration rates between eastern and western populations, contrary to previously inferred patterns from a single mitochondrial locus.

Results for the competing hypotheses of two distinct lineages versus a single‐lineage model of *C. atrox* are shown in Table 2. We found very strong (decisive; Kass & Raftery, 1995) support for the “3 species” model, which included *C. scutulatus* as the outgroup + two parental lineages of *C. atrox*, consistent with previous mtDNA inferences (Castoe et al., 2007; Schield et al., 2015) and demographic model selection in δaδi. Collectively, these inferences support two distinct ancestral/parental lineages of *C. atrox*. STRUCTURE analysis yielded a best‐fit model of *K *=* *2 ancestral populations, which was supported using the Δ*K* method (Δ*K*[2] = 192.7, while Δ*K*[3] = 129.3; Evanno et al., 2005). We observed high posterior probability assignments to each ancestral population cluster in regions outside of the Chihuahuan region, and a gradient of mixed assignments within this region (Fig. S2). We inferred the greatest degree of admixture (i.e., *Q*‐values near 0.5) within samples near the Continental Divide and in western Texas.

**Table 2:** Results of lineage delimitation using BFD*, testing two competing models with *C. scutulatus* as outgroup

Model	Lineages	ML	Rank	BF
No *atrox* divergence* *+ *scutulatus*	2	−2.978	2	–
Two *atrox* lineages + *scutulatus*	3	−2.810	1	−336

ML, marginal likelihood; BF, Bayes factor.

### 3.3 Patterns of genetic differentiation between parental lineages

**Figure 3:**
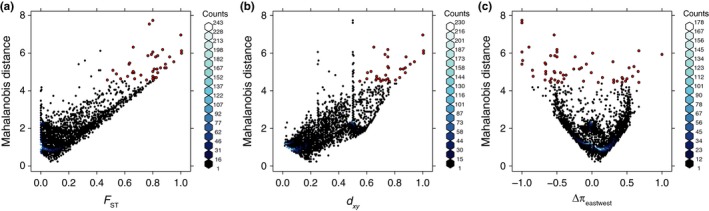
Variation in allelic differentiation between parental eastern and western populations of *C. atrox*. Each comparison depicts the distribution of one univariate measure of differentiation compared to the locus‐specific Mahalanobis distance. (a) Relative differentiation, *F*_ST_. (b)Absolute differentiation, d_*xy*_. (c) Relative nucleotide diversity between eastern and western populations, Δπ_eastwest_. Scales to the right of each panel represent the distribution of bins that quantities of loci fell into within each distribution. Large, red points depict strong statistical outliers

Locus‐specific estimates of genetic diversity and differentiation between parental eastern and western populations varied considerably (Figure 3). Most loci exhibited low relative differentiation between populations (mean *F*_ST_ = 0.09, median *F*_ST_ = 0.022); however, the range of estimates included a number of SNPs with high *F*_ST_ values (Figure 3a), including 10 SNPs (0.14%) with fixed differences (i.e., *F*_ST_ = 1). We also observed 28 SNPs (0.39%) with *F*_ST_ estimates >0.8, and 216 (3.07%) >0.5—values considered consistent with a high degree of population differentiation (Hartl & Clark, 1997). The distribution of absolute allelic differentiation, d_*xy*_, between parental populations was generally lower, as expected, but largely consistent with the *F*_ST_ distribution (Figure 3b). While there was not a perfect linear relationship between genomewide estimates of these two parameters per locus (*r *=* *.58), loci with extreme *F*_ST_ tended to also have extreme values of d_*xy*_. For example, loci with fixed differences estimated using *F*_ST_ also had fixed absolute differentiation (d_*xy*_ = 1). Relative nucleotide diversity (Δπ_eastwest_) ranged from −1 to 1; however, estimates near zero were observed much more frequently (mean = 0.055, median = 0.041), and the 95% confidence interval of Δπ_eastwest_ ranged from −0.42 to 0.48.

We detected 299 statistical outliers and 60 strong statistical outliers that exceeded the 95th and 99th quantiles of the multivariate Mahalanobis distance (MD), respectively. Statistical outliers were associated with elevated estimates of allelic differentiation relative to the genomewide distribution (i.e., minimum *F*_ST_ = 0.16, minimum d_*xy*_ = 0.11), and strong statistical outliers had a minimum *F*_ST_ = 0.27 and a minimum d_*xy*_ = 0.11. We note that some loci with no evidence of allelic differentiation between populations had high MD values; loci could exhibit this pattern due to balancing selection, where shared intermediate frequency alleles are maintained by selection and are expected to result in low *F*_ST_ estimates. Given our inability to determine this explicitly, we removed such loci (with low *F*_ST_ estimates) from comparative analyses of outlier loci.

To examine the fit of our empirical data to purely neutral expectations, we binned empirical *F*_*ST*_ and d_*xy*_ values above discrete ranges of 0.1 (i.e., 0.0–0.1, 0.1–0.2) and compared relative frequencies of empirically observed values and those from simulations conducted using *GppFst* (Fig. S3). Because results were qualitatively similar for both absolute and relative allelic differentiation, we report here on *F*_ST_ results only. We found that the empirical data included far more extreme *F*_ST_ values (e.g., *F*_ST_ > 0.5) than that expected based on the simulated dataset (Fisher's exact *p *<* *1 × 10^−15^); significantly higher proportions of empirical values were observed at all intervals above *F*_ST_ = 0.2, and this was especially pronounced at very extreme *F*_ST_ values. For example, the empirical dataset had 10‐fold more loci than expected with an *F*_ST_ = 1, which was highly significant compared to expectations derived from the neutral model (computed from simulated distribution in *GppFst*;* p *<* *.0001). Thus, based on comparisons of empirical allelic differentiation and neutral expectations, we consider that proportions of outlier loci with *F*_ST_ > 0.2 are poorly explained by neutral evolution and have more likely been driven by selection. We therefore retained 278 statistical outlier loci that exceeded the *F*_ST_ = 0.2 threshold. All 60 strong statistical outliers met this criterion, and were used in downstream candidate gene tests. Importantly, *GppFst* simulations suggest that, at a locus‐specific scale, even strong outliers likely contain false positives. Specifically, based on our *GppFst* simulations, we estimate that there are roughly equal proportions of true and false positives when all loci with 0.2 < *F*_ST_ < 1 are considered. The proportion of false positives substantially decreased, however, with more extreme values of *F*_ST_ (e.g., 19% estimated false positives at 0.6 < *F*_ST_ < 1).

### 3.4 Patterns of genomic introgression

Locus‐specific introgression also varied widely within the admixed *C. atrox* population (Figure 3a). While most loci exhibited clines consistent with the expectation of neutral introgression between parental populations (i.e., nearly equal hybrid index and probability of parental ancestry), some locus‐specific clines deviated from this pattern considerably. Estimates of the genomic cline center parameter, α, ranged from −1.66 to 1.7. The average hybrid index (genomic background of eastern versus western ancestry) of individuals sampled from the admixed population was 0.462, consistent with a nearly completely admixed genomic background (Figure 3a). Given the hybrid index of an individual, particular loci may deviate from the background because they are dominated by either eastern or western alleles, as indicated by the value of α. Locus estimates of the cline rate parameter, β, were less variable, ranging from −0.011 to 0.012, and the 95% confidence interval for all estimates included zero. Locus‐specific clines were thus estimated predominately based on α, and calculated using a β parameter that did not deviate significantly from zero. We identified 283 outlier loci with evidence of excess ancestry (4.02% of loci) based on the distribution of the α parameter. Of these, 133 loci had evidence of western ancestry and 150 loci had evidence of excess eastern ancestry. Despite a greater number of loci with excess eastern ancestry, this difference was not statistically significant (*p* = .337), indicating that there was no evidence of any strong genomewide directional preference toward alleles from one parental lineage over the other in hybrid individuals. We identified 113 strong statistical outliers using locus‐specific γ‐quantiles, where *n* equals 0.1 (γ_0.1_ outliers; see section 2). We found more statistical outliers with eastern ancestry (70 loci) than western (43 loci), and this difference between the number of eastern and western strong statistical outliers was significant (*p* = .0137).

The results of genomic cline analyses on simulated admixed population data were consistent with expectations of neutral introgression (Fig. S4; see also Gompert & Buerkle, 2011). Under these simulations, the probability of parental ancestry was not expected to deviate significantly from predictions based on the hybrid index. In line with this expectation, we observed zero loci with evidence of excess ancestry and no loci were identified as statistical outliers based on the criteria used for empirical analyses; the most extreme values of α were an order of magnitude smaller than in empirical analyses (max. α = .18, min. α = −.13).

### 3.5 Comparative analyses of genomic introgression and divergence

**Figure 4:**
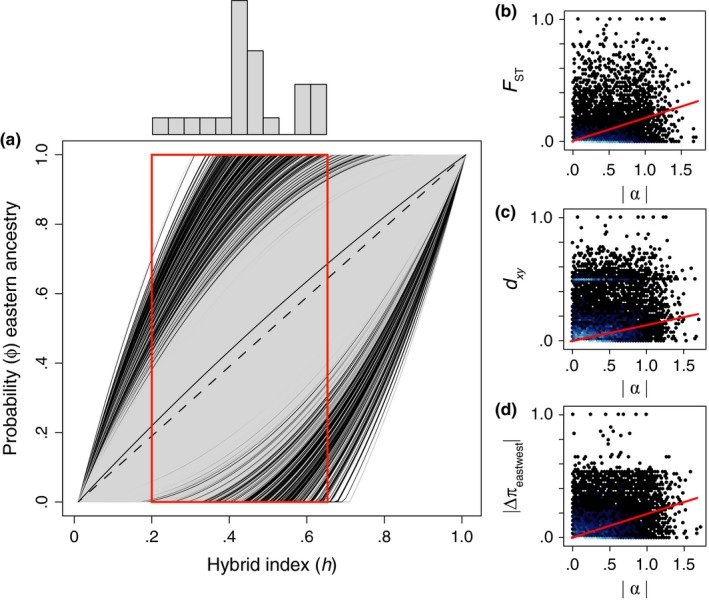
Introgression among genomic loci within hybrid *C. atrox* and depictions of the genomic relationship between allelic differentiation and introgression statistics. (a) Results of genomic cline analysis in *bgc*, depicting the probability of western ancestry given background genomic introgression (i.e., hybrid index) for 7,031 SNP loci. The dashed line represents a perfect linear correlation between hybrid index and ancestry probability as expected under neutral introgression. Clines shown in black represent loci with excess ancestry from one parental population. The histogram above depicts relative frequencies of individual hybrid indices within the admixed population, and the red box is the range of these values on the genomic cline. (b) Comparison of *F*_ST_ with the absolute value of the genomic cline center parameter, α. B. Comparison of d_*xy*_ and the absolute value of α. (c) Comparison of Δπ_eastwest_ and α for all loci. Red lines in (b‐d) represent statistically significant correlations between estimates of differentiation and introgression across the genome. Colors of dots in (b–d) reflect the density of loci, with lighter colors depicting greater quantities of loci

We found positive relationships between locus‐specific measures of allelic differentiation and nucleotide diversity and the genomic cline center parameter α (Figure 4b–d), with linear correlations that were statistically significant. Specifically, the correlations of |α| with *F*_ST_, d_*xy*_, and |Δπ_eastwest_| were as follows: *r* = .22 (*p* = 2.2 × 10^−16^), *r* = .12 (*p* = 2.2 × 10^−16^), and *r* = .22 (*p* = 2.2 × 10^−16^), respectively. We also found a positive relationship between the multivariate Mahalanobis distance from divergence statistics and |α| (*r* = .08, *p* = 2.61 × 10^−9^). Because we found significant, positive correlations between all divergence estimates and introgression, we report further here on *F*_ST_ only. In addition to the overall genomic trend, we observed positive correlations when subsets of loci were considered (e.g., loci with *F*_ST_ > 0.2, *F*_ST_ > 0.5, etc.; Fig. S5). There were a total of 28 loci that were outliers based on both allelic differentiation and excess ancestry in introgression. These contained similar proportions of positive (eastern ancestry; 53.6%) and negative (western ancestry; 46.4%) α values; these proportions were not significantly different (*p* = .82). The same set of loci contained 10 γ_0.1_ outliers with eastern origin and 3 with western origin. Interestingly, however, the most extreme (e.g., top 10) statistical outliers from divergence and introgression analyses were nonoverlapping (unique outlier loci).

### 3.6 Comparative analyses of gene overlap and candidate gene sets

There was higher‐than‐expected overlap in genes putatively linked to loci from divergence and introgression analyses when we compared outliers and strong outliers from these analyses. In both cases, the gene overlap in the empirical dataset exceeded the 95th quantile of overlap observed in resampled datasets (Fig. S6A‐B). It is notable, however, that this is the reverse for the 10 most extreme outliers from each analysis, which did not overlap at all.

**Figure 5:**
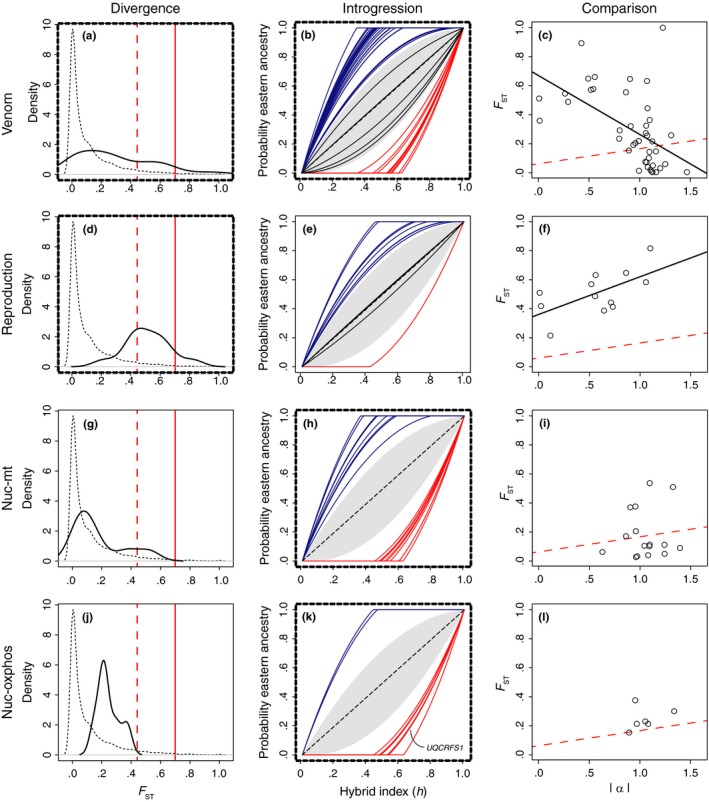
Divergence and introgression of enriched venom (a–c), reproduction (d–f), nuc‐mt (g–i), and nuc‐oxphos (j–l) candidate gene sets. Panels with bold, dashed borders represent outlier locus sets statistically enriched for specific candidate genes. (a, d, g, j) Kernel density plots of candidate gene (black line) and genomewide (dashed line) *F*_ST_ estimates. Red dashed lines represent the 95th *F*_ST_ quantile and solid red lines represent the 99th *F*_ST_ quantile. (b, e, h, k) Genomic clines of candidate genes with excess ancestry. The gray shaded region represents the genome background and is bounded by the positive and negative means of α. Bold colored lines represent outlier locus clines with evidence of excess eastern (blue) and western (red) ancestry. Black clines were candidate gene loci without evidence of excess ancestry. In panel k (nuc‐oxphos genes), the locus‐specific cline for *UQCRFS1* is labeled. (c, f, i, l) Comparisons of locus‐specific |α| and *F*_ST_ values for candidate gene sets. The trend lines in (c) and (f) depict statistically significant correlations. The red dashed lines depict the background genome correlation between *F*_ST_ and |α|, for comparison

Analyses of a priori candidate genes revealed differential patterns of enrichment between sets of genes linked to divergence and introgression outliers (Figure 5; Table S2). Venom genes were enriched in gene sets from both divergence outliers (*p* = .0051) and excess ancestry outliers from introgression (*p* = 7.62 × 10^−5^), but not more stringent divergence and γ _0.1_ outliers, possibly due to low statistical power from small sample sizes (Figure 5a–c). Reproduction candidate genes were enriched in outliers from allelic differentiation (*p* = .0163), but not introgression (Figure 5d–f). There was no evidence for coloration gene enrichment; a single coloration gene was found among excess ancestry outliers, and none were included with divergence outliers. Nuc‐mt and nuc‐oxphos candidate genes exhibited the opposite pattern of reproduction genes (Figure 5g–l), with evidence of enrichment in genes from introgression analysis but no evidence of enrichment in outliers from divergence. Nuc‐mt genes were enriched in both excess ancestry (*p* = .018) and γ_0.1_ introgression outlier genes (*p* = .0097), and nuc‐oxphos genes were enriched in excess ancestry outliers (*p* = .014). Nuc‐oxphos genes with excess ancestry included several ATPases and a nuclear subunit directly involved in oxidative phosphorylation. Specifically, the genomic cline center parameter estimate for the locus putatively linked to *UQCRFS1* (encoding cytochrome C reductase), a component of the cytochrome b‐c1 complex (complex III), was consistent with western ancestry (α = −1.071, 95% CI = −1.792 to −0.302; Figure 5k).

The relationship between measures of allelic differentiation and introgression for candidate gene sets also varied, and in some cases differed considerably from the overall genomic pattern. *F*_ST_ and |α| were negatively correlated for venom genes (*r* = −.53, *p* = .00023; Figure 5c). In contrast, there was a positive correlation between *F*_ST_ and |α| for reproduction genes (*r* = .62, *p* = .032; Figure 5f), which was somewhat surprising given a lack of evidence for enrichment for reproduction in introgression outliers. Finally, we did not find significant correlations between *F*_ST_ and |α| for the nuc‐mt and nuc‐oxphos candidate gene sets, but the trend was toward lower *F*_ST_ distributions (Figure 5g, j) associated with high values of α, which is consistent with evidence for enrichment only in introgression.

## 4 Discussion

### 4.1 Maintenance of lineage integrity upon secondary contact

In this study, we found evidence consistent with previous inferences that populations of *C. atrox* were historically isolated and have more recently experienced secondary contact with hybridization (Tables 1 and 2, Figures 2; S2). The homogenizing effects of pervasive gene flow upon secondary contact should in theory result in a reversal of the divergence and speciation process (Taylor et al., 2006); however, it is becoming clear from population genomic data from natural systems that this is not always the case. Instead, while much of the genome introgresses freely in hybrids, certain regions may resist gene flow due to selection for or against incoming parental alleles because of a direct or indirect impact on hybrid fitness. In the case of *C. atrox*, the hybrid zone might be considered a “tension zone,” where a potentially small proportion of the genome acts to limit homogenization of lineages through partial reproductive isolation (Gay, Crochet, Bell, & Lenormand, 2008). A comparative dissection of the patterns of allelic variation involved in lineage divergence and lineage convergence in secondary contact may thus provide key insight for reconciling interactions between these processes in speciation, and particularly how selection and stochastic forces shape the relationship between these processes. Further identification of common loci under selection in both scenarios implies that there may be links between local adaptation and the maintenance of lineage integrity upon secondary contact and that traits locally co‐adapted in allopatric divergence may also contribute to reproductive isolation. It is poorly understood to what degree we expect loci to be involved in both processes, however, as diverse aspects of demography, time since divergence, geographically diverse selection pressures, and natural history are likely to be influential. Here, we have explored this relationship in Western Diamondback Rattlesnakes, and discuss below ways in which adaptive evolution in divergence and introgression appears to be both linked and also idiosyncratic.

### 4.2 Evidence for selection in divergence and introgression

To address the major hypotheses in this paper, we were interested in identifying loci with patterns likely driven by selection to determine whether adaptation in divergence and fitness in hybrids are linked. We find a highly heterogeneous genomic landscape of allelic differentiation between parental *C. atrox* populations, similar to that reported in recent studies in other species (Ellegren et al., 2012; Ferchaud & Hansen, 2016; Flaxman, Feder, & Nosil, 2013; Nadeau et al., 2012). This distribution included loci with extreme allelic differentiation that are likely linked to genomic regions under selection. Because extreme differentiation could evolve due to stochastic (i.e., drift) or deterministic processes, we tested for signatures of selection‐driven evolution, and employed a novel simulation method (*GppFst*; Adams et al., 2016) to compare empirical distributions of allelic differentiation between parental populations to distributions expected under a neutral coalescent model (including mutation and drift). We found extreme divergence in neutral simulations to be comparatively rare relative to the empirical distribution, suggesting that outlier loci are enriched for loci under selection (Fig. S3).

Even setting cutoffs to highly stringent levels, our simulations suggest that statistical outlier loci are expected to contain false positives, and locus‐specific conclusions should be interpreted with appropriate caution. We note that evolutionary forces not explicitly explored here (e.g., recombination) could also drive false inferences of selection. For example, recombination hotspots may mimic patterns of extreme differentiation due to selection (Myers, Bottolo, Freeman, McVean, & Donnelly, 2005), especially if high recombination rates are paired with rapid mutation rates or biased gene conversion, as has been observed in humans (Lachance & Tishkoff, 2014) and *Drosophila* (Kulathinal, Bennett, Fitzpatrick, & Noor, 2008). Alternatively, low recombination paired with background selection or neutral divergence could drive differentiation between populations (O'Reilly, Birney, & Balding, 2008). With a single pairwise comparison of two populations/lineages in this study, our ability to explicitly account for recombination is limited. However, evidence for biological links between inferred selected loci and enrichment of a priori candidate genes underlying major differences between *C. atrox* populations argues that false positives have not substantially hampered our ability to detect patterns of variation that we expect have been driven by adaptation. Nonetheless, further investigation using information from multiple lineage pairs would be valuable for distinguishing between patterns driven by recombination and “selective sweeps”.

As in divergence analyses, we found the genomewide landscape of introgression in hybrid *C. atrox* to vary considerably, containing extreme locus‐specific clines far outside the expectations of neutral introgression (Fig. S4). For these loci, we propose that selection has acted to resist gene flow to either maintain or resist alleles from one parental population more than would be expected based on the presumably neutral background genomic pattern (Figure 4a; Gompert, Parchman, & Buerkle, 2012b). Loci may fit these extreme patterns because alleles from one parental population offer an increase in hybrid fitness (Payseur, 2010), or because alleles from one parental population are selected against because they are incompatible with the opposite parental genomic background (i.e., Dobzhansky–Muller incompatibilities; Dobzhansky, 1936; Muller, 1942; Orr, 1995).

### 4.3 Genomic relationship between divergence and introgression in secondary contact

The comparison of allelic variation in divergence and introgression revealed an intriguingly complex association across the genome, and broadly identified genomic regions with elevated genetic differentiation between parental *C. atrox* populations that also appear to be under selection in hybrids. Overall, this relationship supports the hypothesis that selection‐driven genomic changes in divergence may also play a role in reproductive isolation in secondary contact, and may further be central to understanding why incipient species may not completely homogenize when they enter secondary contact. This comparison allows us to quantify how tightly linked divergence and admixture processes may be in natural systems, and how divergence in turn translates to reproductive isolation.

A positive relationship between locus‐specific measures of allelic differentiation and the absolute value of α has also been reported in recent studies (Gompert et al., 2012a; Nosil et al., 2012; Parchman et al., 2013). Our findings certainly agree that this relationship exists and provide novel insights into this association in several ways. First, previous studies have specifically considered patterns of variation in *Lycaeides* butterflies (Gompert et al., 2012a), *Timema* stick insects (Nosil et al., 2012), and *Manacus* birds (Parchman et al., 2013). The rattlesnake system studied here is distinct in several respects, including its expansive geographic range encompassing diverse habitat and climatic conditions, dispersal capabilities (i.e., flying birds and insects [*Lycaeides*, specifically] versus crawling reptiles), life‐history traits, and inferred levels of divergence between parental populations. Given biological differences relevant to divergence and introgression between rattlesnakes and previously studied taxa, it is remarkable that this relationship is observed consistently across these disparate systems.

Second, it is notable that the association between allelic differentiation and introgression in *C. atrox* was less than would be expected if *F*_ST_ and α were tightly biologically autocorrelated (Figure 4b–d). This was made apparent by the small proportion (0.4%) of loci that were strong outliers in both analyses and in the 10 most extreme outliers from each analysis not overlapping at all, despite greater‐than‐expected overlap in genes linked to outliers overall (Fig. S6; see also Nosil et al., 2012). In other words, the most extreme outliers in divergence were poor predictors of the most extreme outliers in introgression, suggesting that being under strong selection in divergence is not a singular factor driving importance in introgression. Such idiosyncratic patterns of selection were also observed in variable enrichment of candidate gene sets that revealed intriguing distinctions between *C. atrox* and previously studied systems. In particular, loci that were overlapping extreme outliers from both analyses showed remarkably symmetrical patterns of excess ancestry from eastern and western populations. This is in contrast to overlapping outliers in *Lycaeides* and *Timema*, where there was instead a substantial bias toward excess ancestry from a single parental population over the other (Gompert et al., 2012a; Nosil et al., 2012). The symmetry of excess ancestry in outliers from *C. atrox* supports the hypothesis that adaptive changes in both ancestral populations during divergence contribute to reproductive isolation in hybrids.

### 4.4 Biological interpretations of adaptive evolution in divergence and introgression

Our analyses provide exciting evidence linking patterns of genomewide selection in divergence and introgression with phenotypic traits and expected patterns of prior interest in this species. Specifically, we found that multiple candidate gene sets related to known phenotypic and life‐history differences between *C. atrox* populations, along with putative incompatibilities in hybrids, were enriched for selection. Additionally, correlations between allelic differentiation and introgression for candidate genes varied considerably and were sometimes contrary to the overall genomic relationship, further highlighting multifarious patterns of divergent selection and selection against maladaptive alleles in hybrids. For example, the strong negative correlation between divergence and introgression for venom genes (Figure 5c), despite evidence for enrichment for venom in outliers from both analyses, suggests that specific targets of selection can be fairly unique at the locus level between these processes. An intriguing possibility is that this pattern is driven by the extreme toxicity of venom components and the diversity of tissues and physiological functions they disrupt (Mackessy, 2008), which may drive complex interactions between specific venom alleles and the genomic background to prevent or minimize autotoxicity or other deleterious effects. This diversity likely leads to broad variation in the degree of coevolution of venom alleles with other biological systems that protect rattlesnakes from the action of their own venoms (Nahas, Kamiguti, e Silva, de Barros, & Morena, 1983; Nichol, Douglas, & Peck, 1933; Noguchi, 1909). Variation in levels of venom–genome coevolution, together with geographic variation in prey‐specific venom allele effectiveness (Perez, Pichyangkul, & Garcia, 1979), may explain our findings that selection on venom‐linked loci is idiosyncratic between divergence and admixture despite broad enrichment of venom loci as targets of selection in both processes.

Because differences in reproductive output (i.e., size and number of oocytes and follicles) between eastern and western *C. atrox* females (Spencer, 2003) could result from adaptation to local ecological constraints (Caro et al., 2009; Qualls, 1997), we tested the hypothesis that candidate genes involved in reproduction were enriched for selection. Reproduction‐related genes were enriched in divergence outliers, and the distribution of allelic differentiation among these loci was high overall relative to the genomic background (Figure 5d). Although reproduction‐related genes were not statistically enriched in outliers from introgression analysis, they had *F*_ST_ and α estimates that were tightly correlated (Figure 5f), showing the opposite pattern observed for venom and a pattern that is predicted by the genomic relationship between divergence and introgression. Thus, analyses of reproduction gene enrichment provide indirect evidence for divergent reproductive adaptations between parental populations that may also have an effect on hybrid fitness.

Of the candidate gene sets we considered, genes underlying vertebrate coloration and patterning were the only set with no evidence of enrichment in either divergence or introgression. Indeed, we only observed a single “coloration” gene among outlier loci, which had evidence of excess eastern ancestry. We considered multiple possible reasons for this result. The first is that the detection of signatures of adaptation across the genome using RADseq is limited by a small representative sample of loci (Lowry et al., 2016), and thus, we may have lacked power to detect these selection in these genes. Alternative explanations include that neutral processes have shaped differences in coloration between populations of *C. atrox*, or that selection on coloration is highly locally adapted on a very fine geographic scale that our sampling and analyses were not designed to detect (Campbell & Lamar, 2004; Farallo & Forstner, 2012; Klauber, 1956; Sweet, 1985).

Evidence for selection against introgression of nuclear‐encoded genes that coevolve with the mitochondria (i.e., nuc‐mt and nuc‐oxphos genes) supports inferences from our previous studies that found asymmetry in complements of nuclear allelic content paired with eastern and western mitochondria, suggesting this observation is likely due to cytonuclear incompatibilities (Schield et al., 2015). Further, the lack of evidence for divergent selection on nuc‐mt and nuc‐oxphos genes combined with strong selection against introgression in hybrids matches predictions of hybrid incompatibility accumulation (Orr, 1995). We found exceptional introgression at a gene (*UQCRFS1*; encoding cytochrome c reductase) that interacts directly with mitochondrially encoded subunits of the oxidative phosphorylation cascade. Theory and examples from empirical studies predict that a decoupling of the coevolution between this gene and the mitochondrial background via introgression should impact hybrid fitness by producing maladaptive combinations of nuclear and mitochondrial alleles (Burton, Ellison, & Harrison, 2006; Ellison, Burton, & Promislow, 2006; Ellison, Niehuis, & Gadau, 2008; Sloan, Havird, & Sharbrough, 2016). We found strong evidence of excess ancestry at this and other nuclear‐encoded mitochondrial genes, consistent with selection to reduce the decoupling effect of introgression that appears to in turn contribute to the partial reproductive isolation of *C. atrox* lineages in secondary contact.

## 5 Conclusions

Population genomic studies of historically isolated populations that have experienced secondary contact but maintain some level of lineage integrity through partial reproductive isolation provide new insight into this seemingly paradoxical process. They also provide an important and unique indication of the potential roles of selection in the processes of speciation in allopatry, and in the context of continued maintenance of isolation and lineage integrity in secondary contact. We found compelling evidence that, while the landscape of divergence and introgression is complex, a genomewide relationship between these processes supports the hypothesis that divergent selection in allopatry also contributes to the maintenance of lineage integrity upon secondary contact. In addition to evidence for selection in sets of genes underlying divergent phenotypes between *C. atrox* populations, we find evidence for an important role of loci that have diverged neutrally yet lead to incompatibilities in hybrids (e.g., genes involved in oxidative phosphorylation). Further studies examining secondary contact zones between lineages, particularly lineages with varying levels of divergence, will provide valuable extensions to the work presented here to test the evolutionary replicability and generality of such patterns across species and the speciation continuum.

## Data Accessibility

Genomic data from this study can be found at the NCBI SRA under the bioproject accession PRJNA269607.

## Author Contributions

DRS and TAC conceived and designed the study. DRS and DCC performed molecular laboratory work and prepared sequencing libraries. DRS, RHA, DCC, BWP, GMP, TJ, DMP, ALA, and TAC designed and implemented sequence data analyses. CLS, EES, MKF, and SPM provided intellectual contributions for biological interpretations of results. DRS and TAC wrote the manuscript, and all authors edited the final manuscript.

## Conflict of Interest

None declared.
