# Supplementary material for: Insight into the roles of selection in speciation from genomic patterns of divergence and introgression in secondary contact in venomous rattlesnakes
Source: Ecol Evol. 2017 Apr 23;7(11):3951–66. doi: 10.1002/ece3.2996 (PMC5468163; doi:10.1002/ece3.2996)
Supplement: Supplementary file 1 [file ECE3-7-3951-s001.docx]

Insight into the roles of selection in speciation from genomic patterns of divergence and introgression in secondary contact in venomous rattlesnakes

Appendix I – supplementary methods

### RADseq data generation

We extracted genomic DNA using phenol-chloroform isoamyl alcohol extractions, quantified purified DNA products using a Qubit flourometer (Life Technologies, Grand Island, NY, USA), and loaded DNA into restriction digestion reactions. DNA was digested using Sau3AI and SbfI restriction enzymes and digested products were purified using AMPure beads (Invitrogen, Calsbad, CA, USA). Custom Illumina adapters with dual indices and an 8bp unique molecular identifier (UMI) sequence were then ligated to digested fragments. We size selected for fragments between 575-655 bp using a Blue Pippin Prep (Sage Science, Beverly, MA, USA), targeting roughly 20,000 genomic loci. Size-selected libraries were PCR amplified using barcoded primers and Phusion high-fidelity polymerase enzyme (New England Biolabs, Ipswich, MA, USA), purified using AMPure beads, and quantified using an Agilent Bioanalyzer (Agilent Technologies, Santa Clara, CA, USA). Following quantification, libraries were pooled together in equimolar ratios and sequenced on an Illumina HiSeq 2500 using 100 bp paired-end reads.

### Posterior predictive simulation of population divergence under neutrality

We performed posterior predictive simulations (PPS) of population divergence under a strictly neutral model of mutation and drift using our software *GppFst*. To conduct PPS analysis of genome-wide allelic differentiation, we used 7,031 nuclear SNPs to estimate divergence time and population parameters for the parental eastern and western populations (*τ*_east-west_, θ_west_, θ_east_, and θ_east-west_) via Markov Chain Monte Carlo (MCMC) sampling implemented in the program SNAPP (Bryant et al. 2012). The mean posterior estimates of population genetic parameters used for *GppFst* simulations from SNAPP were as follows: θ_east_ = 0.217, θ_west_ = 0.0592, θ_east-west_ = 0.0615, and *τ*_east-west_ = 0.00578. We ran the MCMC chain for a total of 500,000 generations, sampling every 1,000 generations. We assessed posterior convergence and stationarity using Tracer (for all parameters ESS > 500; (Drummond and Rambaut 2007) and discarded the first 125,000 (25%) steps as burn-in, leaving total of 375 MCMC samples used to generate a PPS *F_ST_* distribution. For each MCMC step, we then simulated 100 independent loci with a length of 190 base pairs under a JC69 model using the R package phybase (Liu and Yu 2010) with random sampling of individuals from the empirical distributions of locus coverage for east and west samples, respectively. We calculated *F*_ST_ and *d_xy_* for a randomly sampled polymorphic site for each simulated locus to generate a theoretical distribution of values, which allowed us to compare empirical distributions of *F*_ST_ and *d_xy_* to identify loci that are poorly explained by the neutral model of divergence. Within this framework, we effectively accounted for multiple sources of uncertainty that may influence our empirical distributions of allelic differentiation, including divergence times, differences in θ parameters for both parental populations, variation in coverage across loci and between populations, and SNP ascertainment.

### Bayesian genomic clines analysis

We ran *bgc* (Gompert and Buerkle 2012) using the genotype uncertainty model using four chains of 70,000 generations each, discarding 40,000 generations as burn-in, and recording parameter estimates from every fifth MCMC generation. Default settings for bgc were used, assuming free recombination between loci, and we set the sequence error probability parameter to 0.001. Parameter estimates from four chains were combined after confirming convergence and stationarity of parameters from each MCMC run. These specifications were used also for simulations of neutral introgression between randomly generated ‘admixed’ loci between eastern and western parental populations (see Methods).

### Candidate gene set construction

To identify regions with homology to known snake venom genes, we used sequences for 22 representative venom gene families from (Reyes-Velasco et al. 2015), to construct a blast database and searched all Cobra genome CDS sequences for all unique coding regions found up and downstream of variant regions, and performed a tblastx search to find putative venom gene family homologs, setting an e-value cutoff of 0.01. We used a similar methodology for coloration candidate genes, for which we obtained sequence for 28 genes involved in vertebrate chromatophores and color patterning (Hoekstra 2006; Hubbard et al. 2010; Irion et al. 2016). We curated a list of ‘reproduction’ candidate genes by combining gene sequences included in the ‘regulation of oocyte development’ and ‘ovarian follicle development’ gene ontology terms (528 total genes). We limited our search to these terms because they were the most closely related to the specific differences in reproductive output between populations of *C. atrox* (Spencer 2003). For the nuclear-encoded mitochondrial gene (nuc-mt) sequence set, we obtained all *Homo sapiens* sequences available in the MitoRes database (1063 genes;(Catalano et al. 2006)). Separately, we generated the more specific oxidative phosphorylation (oxphos) set using genes in the KEGG ‘oxidative phosphrylation’ pathway (120 genes; http://www.genome.jp/dbget-bin/get_linkdb?-t+genes+path:hsa00190), excluding genes encoded in the mitochondrial genome. We generated blast databases for each candidate sequence set, and performed tblastx searches against all up and downstream Cobra genes. For each search, we filtered results to retain a maximum of one target sequence per Cobra gene, and called putative homologs using the e-value distribution, evaluating the point at which 1 – e-value plateaued, indicating consistent sequence similarity among all putative homologs to subject sequences. We tested for enrichment by determining if proportions of candidate genes were greater in outlier sets relative to all Cobra genes, and used Fisher’s Exact Tests to determine if proportions differed significantly.

*Literature Cited*

Bryant, D., R. Bouckaert, J. Felsenstein, N. A. Rosenberg, and A. RoyChoudhury. 2012. Inferring Species Trees Directly from Biallelic Genetic Markers: Bypassing Gene Trees in a Full Coalescent Analysis. Mol Biol Evol 29:1917-1932.

Catalano, D., F. Licciulli, A. Turi, G. Grillo, C. Saccone, and D. D'Elia. 2006. MitoRes: a resource of nuclear-encoded mitochondrial genes and their products in Metazoa. BMC Bioinformatics 7:36.

Drummond, A. J. and A. Rambaut. 2007. BEAST: Bayesian evolutionary analysis by sampling trees. Bmc Evol Biol 7.

Gompert, Z. and C. A. Buerkle. 2012. bgc: Software for Bayesian estimation of genomic clines. Molecular ecology resources 12:1168-1176.

Hoekstra, H. E. 2006. Genetics, development and evolution of adaptive pigmentation in vertebrates. Heredity 97:222-234.

Hubbard, J. K., J. A. Uy, M. E. Hauber, H. E. Hoekstra, and R. J. Safran. 2010. Vertebrate pigmentation: from underlying genes to adaptive function. Trends Genet 26:231-239.

Irion, U., A. P. Singh, and C. Nusslein-Volhard. 2016. The Developmental Genetics of Vertebrate Color Pattern Formation: Lessons from Zebrafish. Current topics in developmental biology 117:141-169.

Liu, L. and L. Yu. 2010. Phybase: an R package for species tree analysis. Bioinformatics 26:962-963.

Reyes-Velasco, J., D. C. Card, A. L. Andrew, K. J. Shaney, R. H. Adams, D. R. Schield, N. R. Casewell, S. P. Mackessy, and T. A. Castoe. 2015. Expression of venom gene homologs in diverse python tissues suggests a new model for the evolution of snake venom. Molecular biology and evolution 32:173-183.

Spencer, C. L. 2003. Geographic variation in morphology, diet, and reproduction of a widespread pitviper, the Western Diamondback Rattlesnake (*Crotalus atrox*). Department of Biology. University of Texas at Arlington, Arlington, TX.
