# Supplementary material for: Insight into the roles of selection in speciation from genomic patterns of divergence and introgression in secondary contact in venomous rattlesnakes
Source: Ecol Evol. 2017 Apr 23;7(11):3951–66. doi: 10.1002/ece3.2996 (PMC5468163; doi:10.1002/ece3.2996)
Supplement: Supplementary file 2 [file ECE3-7-3951-s002.docx]

**Supplementary Online Table 1.** Specimen data for samples used in this study. Where noted, samples were used previously in Schield et al. (2015).

| **Species** | **CA Number** | **Museum ID** | **Voucher** | **Country** | **State** | **County** | **Population** | **Reference** |
| --- | --- | --- | --- | --- | --- | --- | --- | --- |
| *Crotalus atrox* | CA0013 | CAS 235728 | RNF 2596 | USA | CA | Imperial | West | This study |
| *Crotalus atrox* | CA0042 |  | ENT A21 | USA | AZ | Pinal | West | Schield et al. 2015 |
| *Crotalus atrox* | CA0043 |  | ENT A49 | USA | AZ | Pinal | West | Schield et al. 2015 |
| *Crotalus atrox* | CA0046 |  | ENT 11 | USA | AZ | Maricopa | West | Schield et al. 2015 |
| *Crotalus atrox* | CA0048 |  | ENT 7 | USA | AZ | Maricopa | West | Schield et al. 2015 |
| *Crotalus atrox* | CA0049 | LACM 150957 |  | USA | AZ | Pima | West | Schield et al. 2015 |
| *Crotalus atrox* | CA0082 | ROM 18144 |  | USA | CA | Riverside | West | Schield et al. 2015 |
| *Crotalus atrox* | CA0112 | UTAR 50396 | CLS 384 | USA | AZ | Cochise | West | This study |
| *Crotalus atrox* | CA0113 | UTAR 50402 | CLS 388 | USA | AZ | Cochise | West | This study |
| *Crotalus atrox* | CA0179 | UTAR 50405 | CLS 346 | USA | AZ | Cochise | West | This study |
| *Crotalus atrox* | CA0237 |  | CA039 | USA | AZ | Pima | West | This study |
| *Crotalus atrox* | CA0260 |  | CA062 | USA | AZ | Pima | West | This study |
| *Crotalus atrox* | CA0272 |  | CA074 | USA | AZ | Pima | West | This study |
| *Crotalus atrox* | CA0274 |  | CAPR 002 | USA | AZ | Pima | West | This study |
| *Crotalus atrox* | CA0276 |  | CAPR 004 | USA | AZ | Pima | West | This study |
| *Crotalus atrox* | CA0278 |  | CAPR 006 | USA | AZ | Pima | West | This study |
| *Crotalus atrox* | CA0280 |  | CAPR 008 | USA | AZ | Pima | West | This study |
| *Crotalus atrox* | CA0281 |  | CAPR 009 | USA | AZ | Pima | West | This study |
| *Crotalus atrox* | CA0282 |  | CAPR 010 | USA | AZ | Pima | West | This study |
| *Crotalus atrox* | CA0294 |  | CASZ-042 | USA | AZ | Pima | West | This study |
| *Crotalus atrox* | CA0300 |  | Cax001 | USA | AZ | Cochise | West | This study |
| *Crotalus atrox* | CA0304 |  | Cax005 | USA | AZ | Cochise | West | This study |
| *Crotalus atrox* | CA0306 |  | Cax007 | USA | AZ | Santa Cruz | West | This study |
| *Crotalus atrox* | CA0307 |  | Cax008 | USA | AZ | Cochise | West | This study |
| *Crotalus atrox* | CA0308 |  | Cax009 | USA | AZ | Cochise | West | This study |
| *Crotalus atrox* | CA0335 |  | DRS0018 | USA | AZ | Yavapai | West | This study |
| *Crotalus atrox* | CA0345 | NNTRC A29 |  | USA | AZ | Cochise | West | This study |
| *Crotalus atrox* | CA0018 |  | RWV 2001-09 | USA | TX | Jeff Davis | Admixed | Schield et al. 2015 |
| *Crotalus atrox* | CA0021 |  | RWV 2001-13 | USA | NM | Sierra | Admixed | Schield et al. 2015 |
| *Crotalus atrox* | CA0022 |  | RWV 2001-14 | USA | NM | Dona Ana | Admixed | Schield et al. 2015 |
| *Crotalus atrox* | CA0109 | UTAR 50649 | CLS 381 | USA | NM | Hidalgo | Admixed | This study |
| *Crotalus atrox* | CA0110 | UTAR 50445 | CLS 382 | USA | NM | Hidalgo | Admixed | Schield et al. 2015 |
| *Crotalus atrox* | CA0111 | UTAR 50650 | CLS 383 | USA | NM | Hidalgo | Admixed | Schield et al. 2015 |
| *Crotalus atrox* | CA0114 | UTAR 50418 | CLS 393 | USA | NM | Hidalgo | Admixed | This study |
| *Crotalus atrox* | CA0116 | UTAR 50385 | CLS 413 | USA | AZ | Cochise | Admixed | Schield et al. 2015 |
| *Crotalus atrox* | CA0117 | UTAR 50652 | CLS 414 | USA | AZ | Cochise | Admixed | This study |
| *Crotalus atrox* | CA0120 | UTAR 50407 | CLS 419 | USA | AZ | Cochise | Admixed | This study |
| *Crotalus atrox* | CA0130 | UTAR 50409 | CLS 240 | USA | NM | Hidalgo | Admixed | Schield et al. 2015 |
| *Crotalus atrox* | CA0132 | UTAR 50411 | CLS 244 | USA | NM | Hidalgo | Admixed | Schield et al. 2015 |
| *Crotalus atrox* | CA0133 | UTAR 50412 | CLS 247 | USA | NM | Hidalgo | Admixed | This study |
| *Crotalus atrox* | CA0139 | UTAR 50398 | CLS 264 | USA | AZ | Cochise | Admixed | Schield et al. 2015 |
| *Crotalus atrox* | CA0146 | UTAR 50399 | CLS 282 | USA | AZ | Cochise | Admixed | Schield et al. 2015 |
| *Crotalus atrox* | CA0150 | UTAR 50422 | CLS 287 | USA | NM | Hidalgo | Admixed | This study |
| *Crotalus atrox* | CA0151 | UTAR 50376 | CLS 290 | USA | AZ | Cochise | Admixed | Schield et al. 2015 |
| *Crotalus atrox* | CA0158 | UTAR 50428 | CLS 298 | USA | NM | Hidalgo | Admixed | Schield et al. 2015 |
| *Crotalus atrox* | CA0160 | UTAR 50429 | CLS 300 | USA | NM | Hidalgo | Admixed | Schield et al. 2015 |
| *Crotalus atrox* | CA0161 | UTAR 50426 | CLS 301 | USA | NM | Hidalgo | Admixed | This study |
| *Crotalus atrox* | CA0171 | UTAR 50404 | CLS 344 | USA | AZ | Cochise | Admixed | Schield et al. 2015 |
| *Crotalus atrox* | CA0339 |  | DRS0016 | USA | TX | Reeves | Admixed | This study |
| *Crotalus atrox* | CA0028 |  | RWV 2001-22 | USA | TX | Jeff Davis | East | Schield et al. 2015 |
| *Crotalus atrox* | CA0039 |  | BLC 27 | USA | NM | Sierra | East | Schield et al. 2015 |
| *Crotalus atrox* | CA0063 |  | TJL601 | USA | TX | Llano | East | Schield et al. 2015 |
| *Crotalus atrox* | CA0065 |  | TJL868 | USA | TX | Potter | East | Schield et al. 2015 |
| *Crotalus atrox* | CA0068 |  | TJL347 | USA | TX | Val Verde | East | This study |
| *Crotalus atrox* | CA0069 |  | TJL348 | USA | TX | Val Verde | East | Schield et al. 2015 |
| *Crotalus atrox* | CA0073 |  | JJ | USA | TX | Culberson | East | Schield et al. 2015 |
| *Crotalus atrox* | CA0096 |  | RLG381 | USA | TX | LaSalle | East | Schield et al. 2015 |
| *Crotalus atrox* | CA0097 |  | RLG367 | USA | TX | Val Verde | East | Schield et al. 2015 |
| *Crotalus atrox* | CA0098 |  | RLG380 | USA | TX | LaSalle | East | Schield et al. 2015 |
| *Crotalus atrox* | CA0099 |  | RLG390 | USA | TX | Dimmit | East | Schield et al. 2015 |
| *Crotalus atrox* | CA0100 |  | RLG404 | USA | TX | Zavala | East | Schield et al. 2015 |
| *Crotalus atrox* | CA0193 |  | DRS0002 | USA | TX | Shackelford | East | Schield et al. 2015 |
| *Crotalus atrox* | CA0194 |  | DRS0003 | USA | TX | Palo Pinto | East | Schield et al. 2015 |
| *Crotalus atrox* | CA0195 |  | DRS0005 | USA | TX | Parker | East | Schield et al. 2015 |
| *Crotalus atrox* | CA0196 |  | DRS0007 | USA | TX | Tom Green | East | Schield et al. 2015 |
| *Crotalus atrox* | CA0342 | NNTRC A26 |  | USA | TX | Nueces | East | This study |
| *Crotalus atrox* | CA0343 | NNTRC A21 |  | USA | TX | Kleburg | East | This study |
| *Crotalus atrox* | CA0344 | NNTRC A51 |  | USA | TX | Kleburg | East | This study |
| *Crotalus scutulatus* | CS0121 |  | Csx001 | USA | AZ | Graham |  | This study |
| *Crotalus scutulatus* | CS0125 |  | Csx004 | USA | AZ | Cochise |  | This study |
| *Crotalus scutulatus* | CS0126 |  | Csx005a | USA | AZ | Pima |  | This study |
| *Crotalus scutulatus* | CS0130 | CAS 228094 |  | USA | CA | San Bernardino |  | This study |
| *Crotalus scutulatus* | CS0131 |  | CLS 800 | USA | CA | San Bernardino |  | This study |
| *Crotalus scutulatus* | CS0132 |  | CLS 811 | USA | CA | Los Angeles |  | This study |

**Supplementary Online Table 2.** Numbers of putative homologs to venom, reproduction, coloration, nuc-mt, and nuc-oxphos candidate gene sets from all orthologous Cobra genome regions, as well as specific outlier locus sets. Proportions of candidate genes in each outlier locus set and results of Fisher's Exact tests are provided below. Outlier sets in bold were significantly enriched.

| **Set** | **All genes** | **Venom genes** | **Reproduction genes** | **Coloration genes** | **Nuc-mt genes** | | **Nuc-oxphos genes** | |
| --- | --- | --- | --- | --- | --- | --- | --- | --- |
| All loci | 5992 | 265 | 206 | 25 | 199 | 56 | |  |
| Divergence | 190 | 18 | 14 | 0 | 11 | 2 | |  |
| Strong divergence | 38 | 4 | 2 | 0 | 2 | 0 | |  |
| Introgression | 312 | 32 | 12 | 1 | 19 | 8 | |  |
| Strong introgression | 66 | 4 | 3 | 0 | 8 | 1 | |  |

| **Fisher's Exact Test - Venom** | |  |
| --- | --- | --- |
| Set | Prop. venom | p-value |
| All loci | 0.044 | - |
| **Divergence** | **0.095** | **0.005071** |
| Strong divergence | 0.110 | 0.1028 |
| **Introgression** | **0.103** | **7.62E-05** |
| Strong introgression | 0.061 | 0.5418 |
|  |  |  |
| **Fisher's Exact Test - Reproduction** | |  |
| Set | Prop. reproduction | p-value |
| All loci | 0.034 | - |
| **Divergence** | **0.074** | **0.0163** |
| Strong divergence | 0.053 | 0.3874 |
| Introgression | 0.038 | 0.6357 |
| Strong introgression | 0.045 | 0.501 |
|  |  |  |
| **Fisher's Exact Test - Coloration** | |  |
| Set | Prop. coloration | p-value |
| All loci | 0.004 | - |
| Divergence | 0 | 1 |
| Strong divergence | 0 | 1 |
| Introgression | 0.003 | 1 |
| Strong introgression | 0 | 1 |
|  |  |  |
| **Fisher's Exact Test - Nuc-mt** | |  |
| Set | Prop. nuc-mt | p-value |
| All loci | 0.033 | - |
| Divergence | 0.058 | 0.1019 |
| Strong divergence | 0.053 | 0.3717 |
| **Introgression** | **0.061** | **0.01802** |
| **Strong introgression** | **0.121** | **0.009745** |
|  |  |  |
|  |  |  |
| **Fisher's Exact Test - Nuc-oxphos** | |  |
| Set | Prop. nuc-oxphos | pval |
| All loci | 0.009 | - |
| Divergence | 0.011 | 0.6992 |
| Strong divergence | 0 | 1 |
| **Introgression** | **0.026** | **0.01414** |
| Strong introgression | 0.015 | 0.4679 |


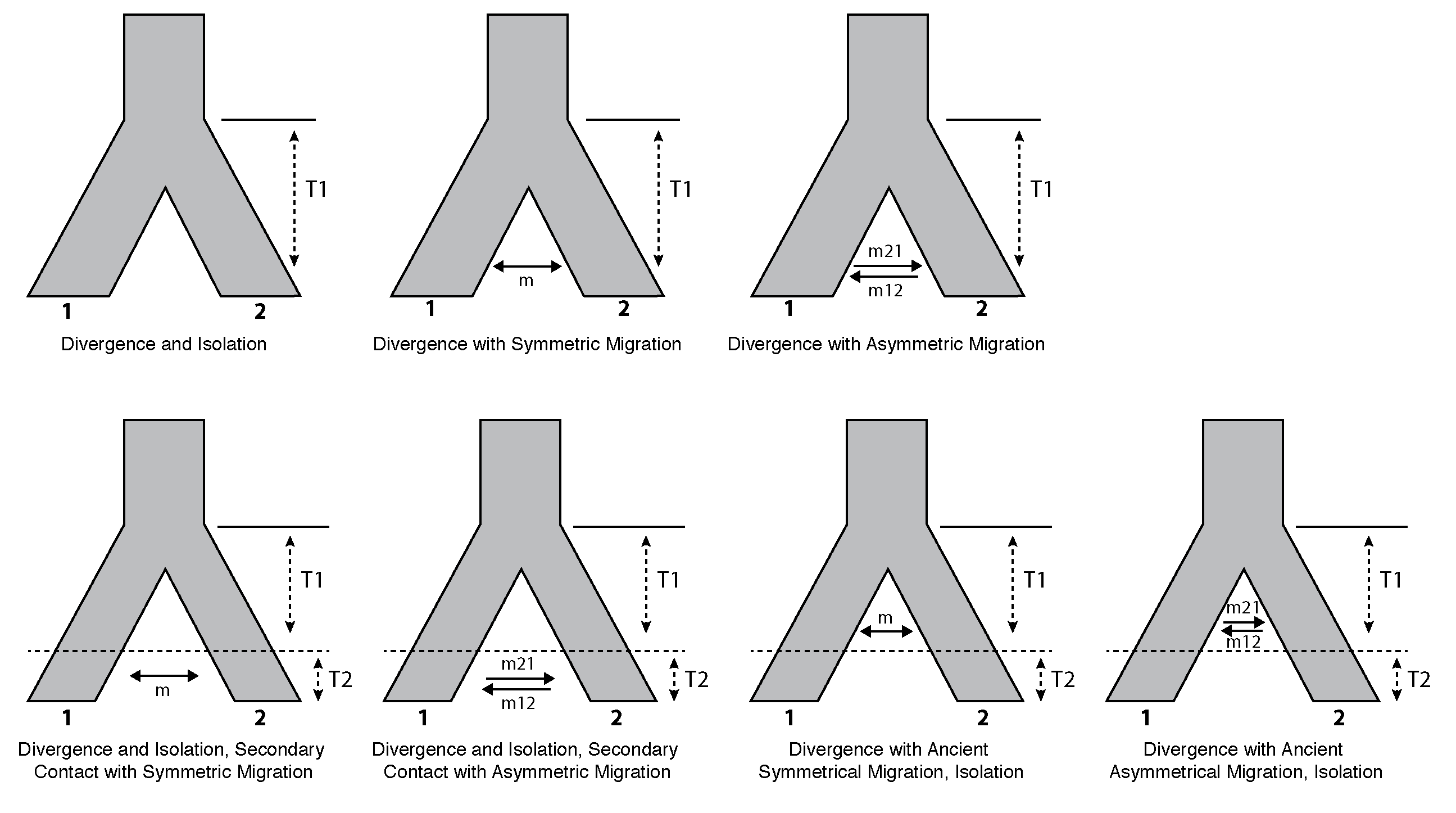


**Supplementary Online Figure 1.** Competing demographic models tested using the 2D allele frequency spectrum between eastern and western populations. Models involving no population divergence are not shown. See Table 1 and Figure 2 for detailed information about parameter estimates and the best-fit model.


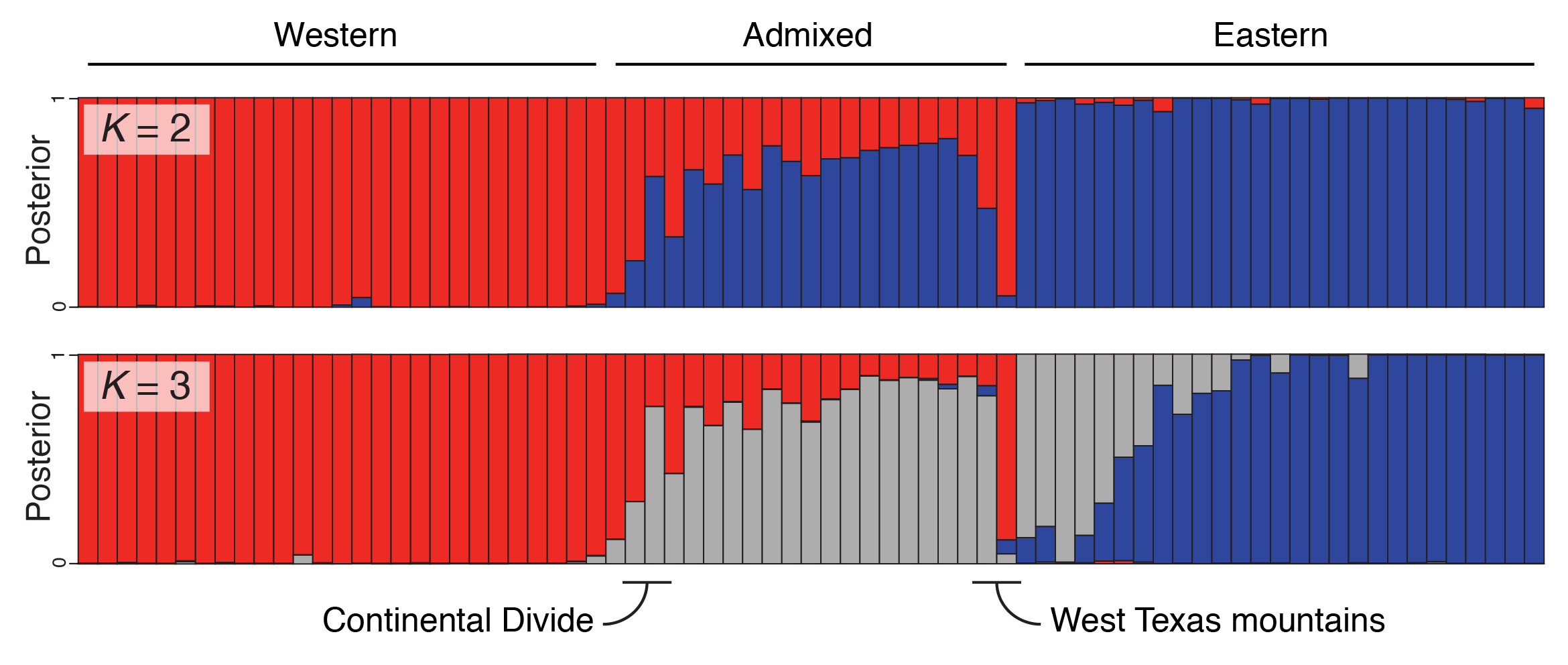


**Supplementary Online Figure 2.** Posterior probability assignments of each individual into genetic clusters inferred using STRUCTURE organized in a longitudinal gradient from west to east under K = 2 and K = 3 models. Geographic assignments used in divergence and introgression analyses are labeled below. The transitions across the Continental Divide and the mountains in western Texas are labeled under the individuals sampled at those localities.


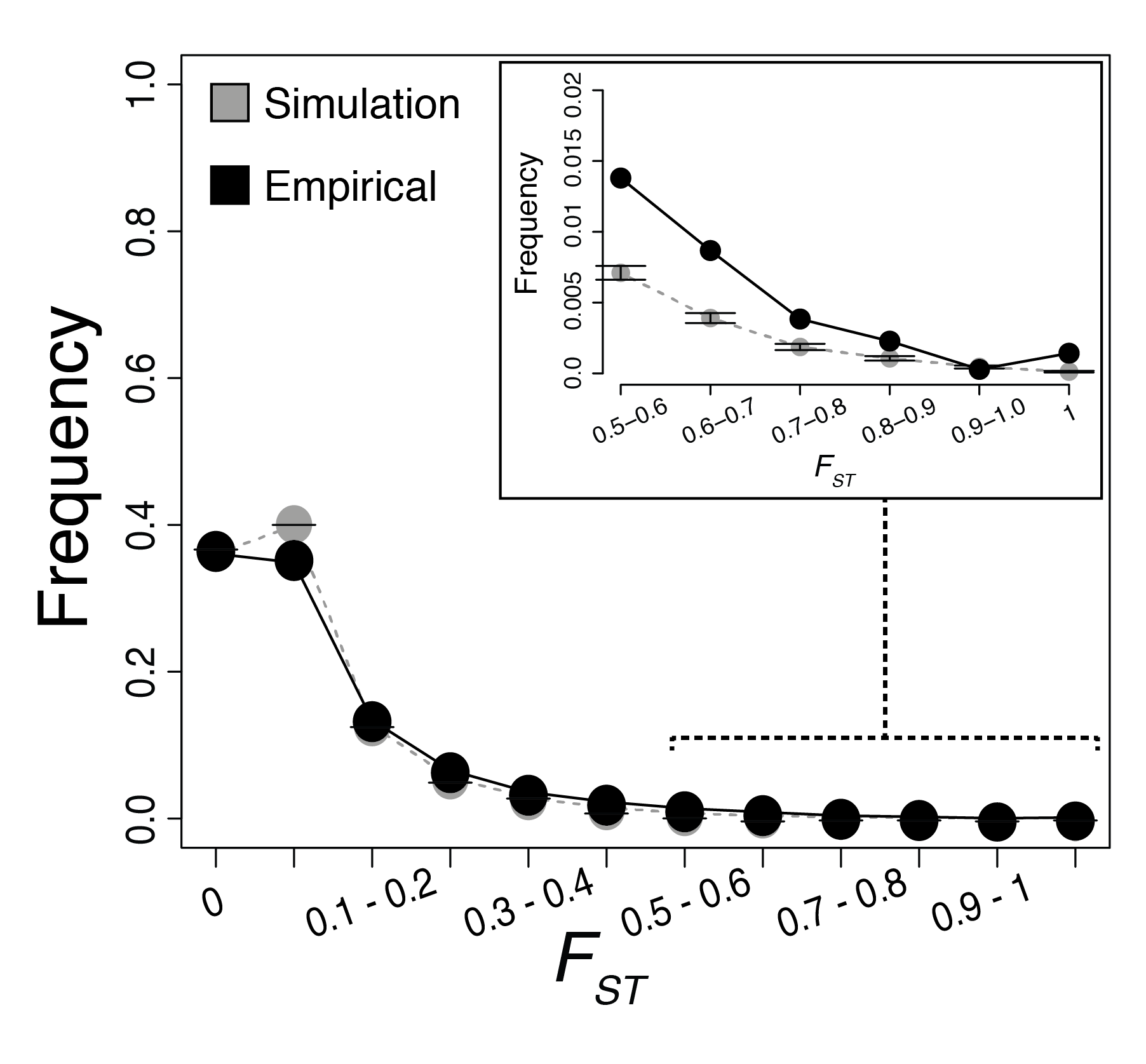


**Supplementary Online Figure 3.** Results of analyses of empirical and simulated distributions of *F*_ST_ in *GppFst*, showing proportions of loci from simulated (grey) and empirical (black) datasets that fall into bins of *F*_ST_ values in 0.1 intervals. The inset details proportions of loci with *F*_ST_ in bins greater than 0.5. Error bars on posterior predictive simulated points indicate the standard deviation of estimates across 100 replicates.


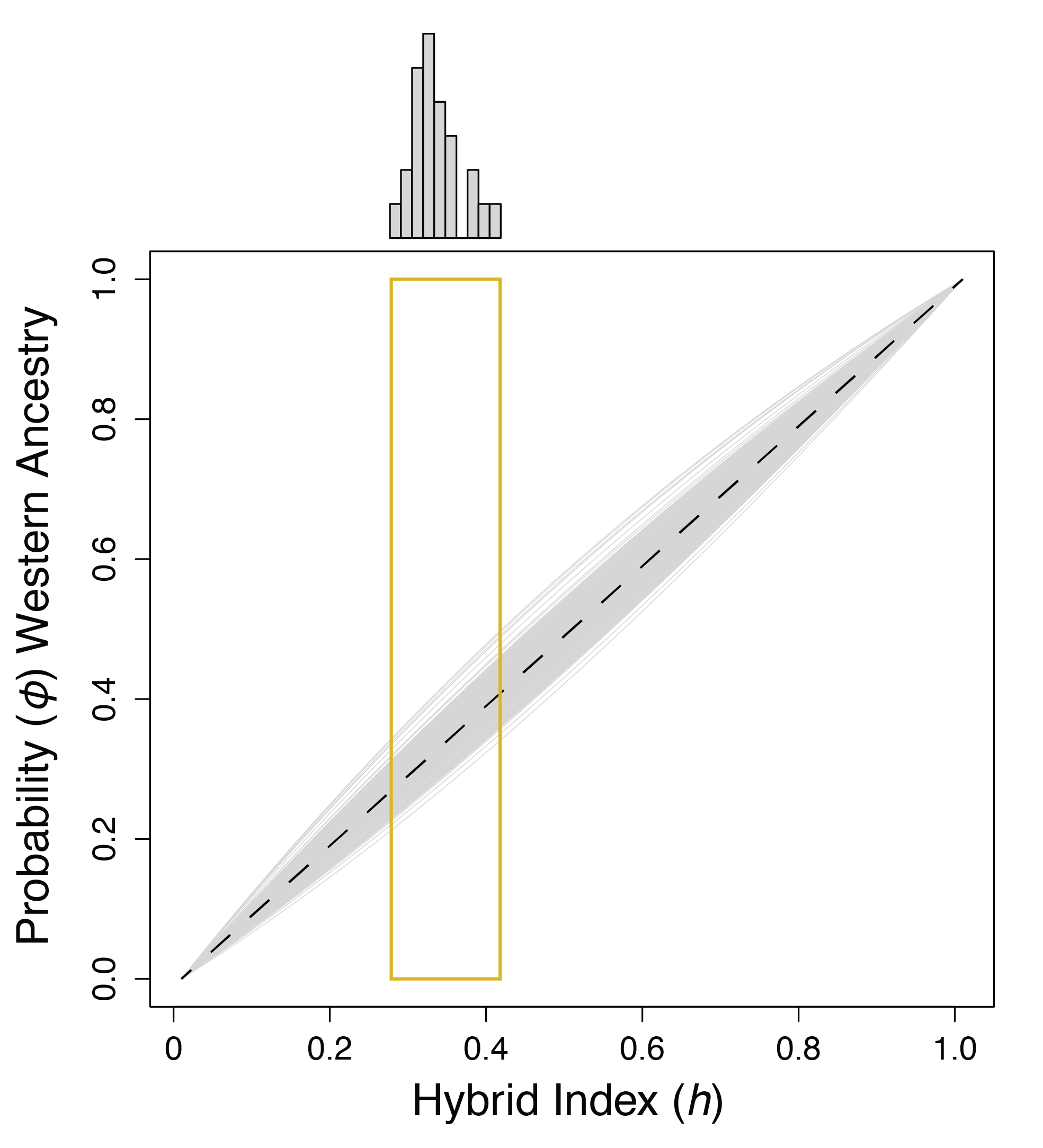


**Supplementary Online Figure 4.** Results of genomic cline analysis using an admixed population simulated from random alleles from each parental population for 7,031 loci. The dashed line represents a perfect linear correlation between hybrid index and ancestry probability as expected under neutral evolution. The histogram above depicts relative frequencies of individual hybrid indices within the admixed population, and the yellow box denotes the range of these values on the genomic cline.

**Supplementary Online Figure 5.** Correlation coefficients between FST and |α| for various intervals of FST. The black line/points indicate comparisons that were made including only loci equal to or greater than the value of the interval. The green line depicts comparisons where only loci with values less than the interval were used.


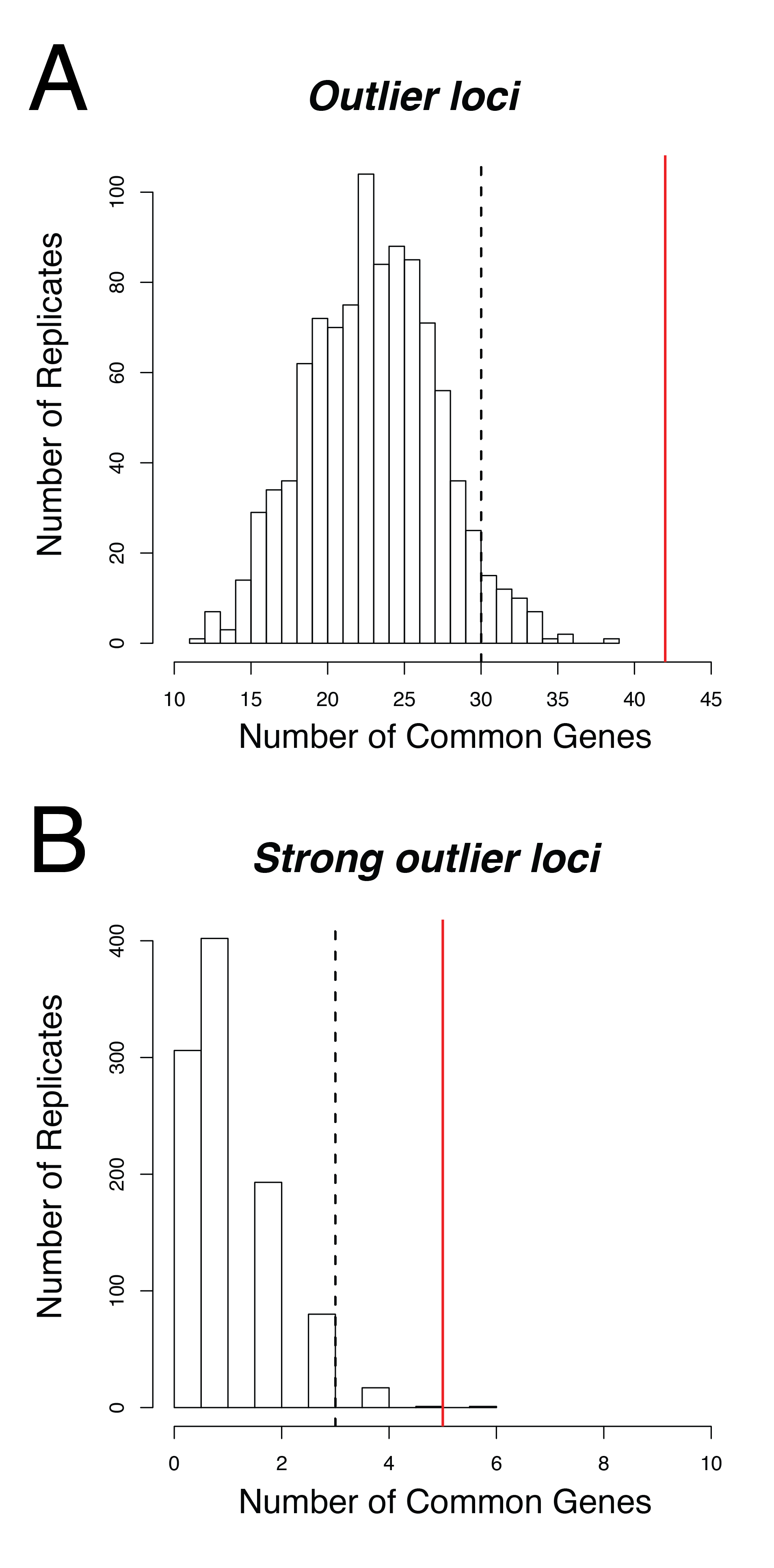


**Supplementary Online Figure 6.** Comparisons of observed numbers of overlapping genes and distributions of random samples of overlapping genes between genes linked to outlier loci (A) and strong outlier loci (B) from divergence and introgression analyses. Red lines denote the number of observed overlapping genes, and dashed black lines indicate the 95th percentile of the random sample distribution.
